# High‐Performance Ideal Bandgap Sn‐Pb Mixed Perovskite Solar Cells Achieved by MXene Passivation

**DOI:** 10.1002/smll.202403920

**Published:** 2024-08-15

**Authors:** Jiupeng Cao, Chun‐ki Liu, Yang Xu, Hok‐Leung Loi, Tianyue Wang, Mitch Guijun Li, Lixian Liu, Feng Yan

**Affiliations:** ^1^ Department of Applied Physics The Hong Kong Polytechnic University Hung Hom Kowloon Hong Kong SAR 999077 P. R. China; ^2^ Division of Integrative Systems and Design Department of Electronic and Computer Engineering The Hong Kong University of Science and Technology Clear Water Bay Kowloon Hong Kong SAR 999077 P. R. China; ^3^ School of Optoelectronic Engineering Xidian University Xi'an 710071 P. R. China; ^4^ Research Institute of Intelligent Wearable Systems The Hong Kong Polytechnic University Hung Hom Kowloon Hong Kong SAR 999077 P. R. China

**Keywords:** 2D perovskite, ideal‐bandgap perovskite solar cells, Mxene, stability

## Abstract

Ideal bandgap (1.3–1.4 eV) Sn‐Pb mixed perovskite solar cells (PSC) hold the maximum theoretical efficiency given by the Shockley–Queisser limit. However, achieving high efficiency and stable Sn‐Pb mixed PSCs remains challenging. Here, piperazine‐1,4‐diium tetrafluoroborate (PDT) is introduced as spacer for bottom interface modification of ideal bandgap Sn‐Pb mixed perovskite. This spacer enhances the quality of the upper perovskite layer and forms better energy band alignment, leading to enhanced charge extraction at the hole transport layer (HTL)/perovskite interface. Then, 2D Ti_3_C_2_T_x_ MXene is incorporated for surface treatment of perovskite, resulting in reduced surface trap density and enhanced interfacial electron transfer. The combinations of double‐sided treatment afford the ideal bandgap PSC with a high efficiency of 20.45% along with improved environment stability. This work provides a feasible guideline to prepare high‐performance and stable ideal‐bandgap PSCs.

## Graphical abstract

**Piperazine‐1,4‐diium tetrafluoroborate** is introduced for bottom interface modification of ideal bandgap Sn‐Pb mixed perovskite, which enhances the quality of the upper perovskite layer and forms better energy band alignment. Furthermore, 2D MXene is incorporated for surface treatment of perovskite on the top to reduce surface trap density and enhance interfacial electron transfer, leading to improved efficiency and stability.

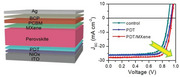

## 1 Introduction

Since 2009, organic–inorganic halide perovskite solar cells (PSC) have achieved great development, with a certified power conversion efficiency (PCE) of 26.7%.^[^ ^1^, ^2^ ^]^ Currently, the most efficient PSCs reported are based on lead‐based perovskites, which have a bandgap of 1.5–1.7 eV. However, the ideal bandgap of single‐junction solar cells should be ≈1.3–1.4 eV according to the Shockley–Queisser (SQ) limit.^[^ ^3^, ^4^, ^5^ ^]^ Bandgap tuning of perovskites by compositional engineering is a facial method to achieve ideal bandgap perovskite light absorbers for single‐junction solar cells. Specifically, ideal‐bandgap perovskite can be realized from Sn‐Pb mixed perovskite with a Sn/Pb ratio of ≈3/7.^[^ ^6^, ^7^ ^]^ Moreover, low bandgap Sn‐Pb mixed perovskites are necessary for all perovskite tandem devices.^[^ ^8^, ^9^, ^10^ ^]^

However, the efficiency of Sn‐Pb mixed PSCs still falls behind that of their lead‐based counterparts, which arises from the high defect density induced by the Sn^2+^ oxidation.^[^ ^10^, ^11^ ^]^ Various efforts have been made to enhance the efficiency and stability of Sn‐Pb mixed PSCs. For example, tin fluoride (SnF_2_) has been widely used as an additive in Sn‐Pb mixed perovskite to suppress the oxidation of Sn^2+^ and enhance the crystallinity of perovskites.^[^ ^12^ ^]^ Recently, Xiao and co‐workers introduced an antioxidant formamidine sulfinic (FSA) additive to suppress the Sn^2+^ oxidation and passivate defects in Sn‐Pb mixed perovskite, and a high efficiency of 21.7% was obtained in low‐bandgap (1.22 eV) Sn‐Pb mixed PSCs.^[^ ^13^ ^]^ Tong et al. reported the incorporation of SnCl_2_·3FACl additive in Sn‐Pb mixed perovskite for enhanced crystallinity and reduced residual stress with promising effects.^[^ ^14^ ^]^ Nevertheless, it is still challenging to prepare high performance ideal‐bandgap Sn‐Pb mixed PSCs with decent long term stability.

In this work, piperazine‐1,4‐diium tetrafluoroborate (PDT) was used to form an interfacial layer at the bottom side of ideal bandgap Sn‐Pb mixed perovskite, which has a composition of FA_0.7_MA_0.2_Cs_0.1_Pb_0.7_Sn_0.3_I_3_ (FA, formamidinium; MA, methylammonium; Cs, cesium). This interfacial layer forms favorable energy alignment between the hole transport layer (HTL) and perovskite, which is beneficial for efficient charge separation and collection. Meanwhile, tetrafluoroborate (BF_4_^−^) at the HTL/perovskite interface can effectively passivate the defects in perovskites to reduce nonradiative recombination.^[^ ^15^ ^]^ Then, we post‐treated the perovskite with 2D Ti_3_C_2_T_x_ MXene. The MXene interlayer can effectively passivate the surface defects and tune the work function of perovskite to form proper energy level alignment at perovskite/electron transport layer (ETL) for enhanced charge transport. Consequently, the ideal bandgap PSCs with dual interfacial modification showed high PCE of 20.45% along with improved long‐term stability.

## 2 Results and Discussion

**Figure 1:**
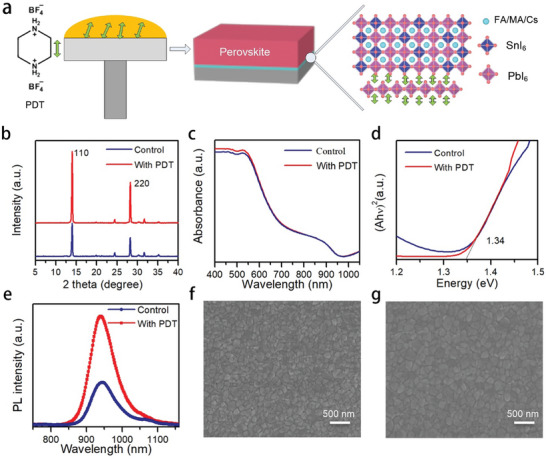
a) Schematic diagram of the fabrication process of PDT interlayer. b) XRD patterns, (b) UV–vis absorption spectra, c) Colcualted bandgap, and e) PL spectra of perovskite film with and without PDT interlayer. SEM images of perovskite film f) without and g) with PDT interlayer.

Schematic illustration of the fabrication process of ideal bandgap Sn‐Pb mixed perovskite based on PDT interfacial layer is shown in **Figure** **1**a, where PDT/PbI_2_ complex was coated on the substrate and then the perovskite precursor was deposited. The X‐ray diffraction (XRD) pattern of the PDT/PbI_2_ complex is shown in Figure S1 (Supporting Information), and the characteristic peak at ≈5° indicates the formation of low dimensional perovskite.^[^ ^16^, ^17^ ^]^ XRD was then used to investigate the crystalline properties of the perovskite films with and without the interlayer. As can be seen from Figure 1b, the diffraction peaks ≈14° and 28° can be ascribed to the (110) and (220) lattice planes of FA_0.7_MA_0.2_Cs_0.1_Pb_0.7_Sn_0.3_I_3_ crystal, respectively.^[^ ^18^ ^]^ With the introduction of the interlayer, the diffraction peak shows a smaller full width at half maximum (FWHM), which indicates the enhanced crystallinity of the perovskite film.^[^ ^19^ ^]^ Meanwhile, there is no obvious peak appearing below 10°, which can be ascribed to the weak diffraction intensity of the ultrathin interlayer.

The absorption spectra of the perovskite films are shown in Figure 1c, which show similar absorbing edges. Figure 1d shows the bandgap (E_g_) of the perovskite films (1.34 eV), which is close to the ideal bandgap value of the SQ limit. Figure 1e shows the steady photoluminescence (PL) spectra of the perovskite films. With the introduction of the interlayer, the perovskite shows stronger PL intensity, which indicates reduced nonradiative recombination. Specifically, BF_4_^−^ can decrease the interstitial iodide defects and thus reduce nonradiative recombination.^[^ ^15^, ^20^ ^]^ To study the effects of the interlayer on the morphology of the perovskite films, scanning electron microscope (SEM) images are taken and shown in Figure 1f,g. With the introduction of the interlayer, the grain size of the perovskite film is enlarged and the perovskite film is more compact. Then, a lift‐off method was used to investigate the bottom interface morphology of the perovskite films.^[^ ^21^ ^]^ As shown in Figure S2 (Supporting Information), some voids can be seen from the control sample while these voids were greatly decreased in the interlayer modified perovskite film. The interlayer may act as a template to assist the growth of perovskite grains, leading to improved perovskite morphology with larger grain size.^[^ ^22^ ^]^

**Figure 2:**
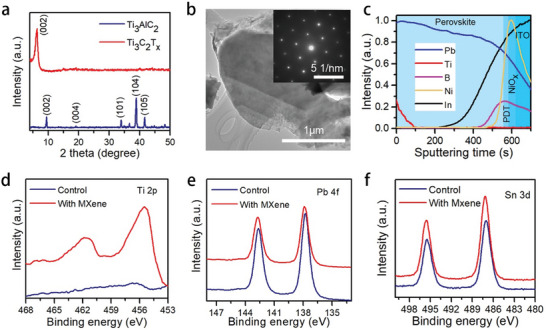
a) XRD patterns of Ti_3_AlC_2_ and Ti_3_C_2_T_x_ MXene. b) TEM image of Ti_3_C_2_T_x_ MXene and the inset shows the corresponding SAED pattern. c) TOF‐SIMS profile of MXene/perovskite/PDT/NiO_x_/ITO. d) Ti 2p, e) Pb 4f, and f) Sn 3d XPS spectra of perovskite film with and without MXene treatment.

MXene is a new type of 2D material with excellent optoelectric properties like high electrical conductivity and large specific surface.^[^ ^23^ ^]^ Moreover, the surface groups (oxygen (‐O), hydroxyl (‐OH), and fluorine‐containing groups (‐F)) of MXene can tune the electronic properties,^[^ ^24^ ^]^ making MXene promising material for application in PSCs.^[^ ^25^ ^]^ Here, Ti_3_AlC_2_ powder was etched to remove the Al layers to get the Ti_3_C_2_T_x_ MXene nanosheets. XRD patterns of Ti_3_AlC_2_ before and after etching are shown in **Figure** **2**a. Disappearance of the characteristic (104) peak of Ti_3_AlC_2_ at 39° and the shift of (002) peak indicate the synthesis of Ti_3_C_2_T_x_ nanosheets.^[^ ^26^ ^]^ As shown in Figure 2b, the transmission electron microscopy (TEM) image shows the few layered nanosheets with lateral size of ≈1 µm. The selected area electron diffraction (SAED) in Figure 2b is consistent with hexagonal atomic structure. The average size of the MXene nanosheets measured by dynamic light scattering (DLS) was ≈900 nm (Figure S3, Supporting Information). The thickness of the MXene nanosheets was determined using atomic force microscopy (AFM), and the thickness is ≈2 nm (Figure S4, Supporting Information). The high‐resolution TEM (HRTEM) of MXene nanoflakes is shown in Figure S5 (Supporting Information), and the lattice fringe spacing of 0.26 nm can be ascribed to the (101) plane.^[^ ^27^ ^]^

Subsequently, the high quality MXene with functional groups was used to post‐treat the perovskite films. Time of flight secondary ion mass spectroscopy (TOF‐SIMS) was used to investigate the cross‐sectional elements distribution. As illustrated in Figure 2c, appearance of Ti ions confirmed the existence of MXene on the perovskite film. Meanwhile, the distribution of B ions near the bottom of perovskite indicates the formation of the PDT interlayer, leading reduced trap density.^[^ ^20^, ^28^ ^]^ XPS was further carried out to investigate the interaction between MXene and perovskite, and the XPS spectra are shown in Figure 2d–f. The Ti peak in the MXene‐modified perovskite further confirmed the presence of MXene on the perovskite film. As illustrated in Figure 2e,f, the binding energies of Sn and Pb show an obvious shift after MXene treatment, which indicates the chemical interaction between MXene and perovskite.^[^ ^29^ ^]^ This interaction suggests the functional groups of MXene (‐OH, ‐F, and ‐O) can interact with undercoordinated Pb^2+^/Sn^2+^ to reduce the defect density of the perovskite film.^[^ ^30^, ^31^, ^32^ ^]^

**Figure 3:**
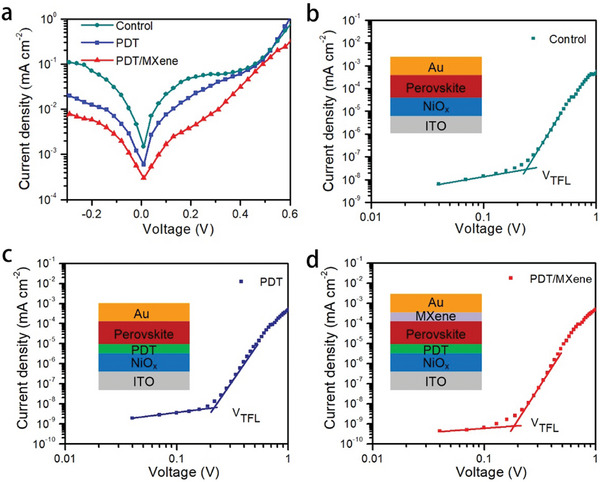
a) Dark *J–V* curves of the perovskite films. Dark *J–V* curves of hole‐only devices b) control, c) PDT modified and d) PDT/MXene modified.

To evaluate the effect of PDT/MXene treatment on the charge transport properties,^[^ ^33^ ^]^ dark current density was measured and shown in **Figure** **3**a. The plain device suffered from high leakage current. In contrast, the PDT/MXene double‐sided treated device showed good diode behavior with much lower leakage current, indicating the much reduced background carrier density. Since the background carrier density is related with the trap density in a solar cell, this results suggest the double‐sided treatment can decrease the trap density in the devices.^[^ ^34^ ^]^ To quantitatively estimate the trap density, the space charge limited current (SCLC) measurement was performed. Hole‐only devices were prepared and dark *I–V* curves were measured and shown in Figure 3b–d. The Ohmic region at low bias exhibited a linear relation between current and electric field. When the voltage is above the trap‐filled limit voltage *V_TFL_*, the current shows a sharp increase. The trap density can be calculated from the equation of *N_t_* = (2εε_0_ *V_TFL_*)/(eL^2^), where ε and ε_0_ is the dielectric constant of perovskite and vacuum dielectric constant, respectively. L is the thickness of perovskite and e is the elementary charge.^[^ ^35^ ^]^ PDT/MXene double‐sided modification leads to decreased defect density from 8.0 × 10^14^ to 6.1 × 10^14^ cm^−3^, which validates that the double‐sided treatment can effectively reduce trap states in perovskite films. As discussed above, BF_4_^−^ ions can enhance the film quality and reduce interstitial iodide defects. Meanwhile, the MXene post‐treatment can effectively passivate interfacial defects through the chemical interactions. In addition, to get insight into the carrier recombination behavior inside the devices, open voltage (*V_OC_*) of the devices under various light intensities was measured and shown in Figure S6 (Supporting Information). The dominant recombination mechanism in a solar cell can be reflected from the ideality factor *n*.^[^ ^36^ ^]^ Generally, the value of *n* closer to 1 suggests less defect‐induced recombination.^[^ ^37^ ^]^ The target device has much lower *n* value than that of the control device, indicating the suppressed nonradiative recombination.

**Figure 4:**
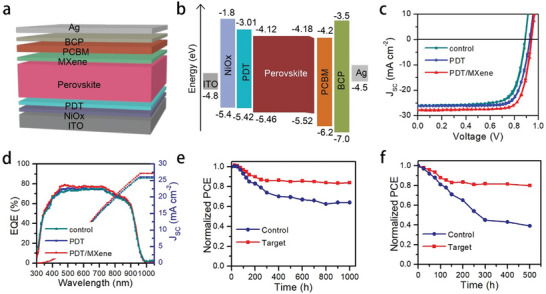
a) Device structure of ideal‐bandgap PSC. b) Schematic energy level of the PSC. c) *J–V* curves and d) EQE spectra of the PSC without and with interlayer modification. Long‐term stability of the unencapsulated device under e) N_2_ glove box and f) white light illumination in N_2_ glove box.

PSCs with a structure of ITO/NiO_x_/PDT/perovskite/MXene/PCBM/BCP/Ag are prepared (**Figure** **4**a). Cross‐sectional SEM image of the device is shown in Figure S7 (Supporting Information), and the thickness of perovskite layer is ≈550 nm. UV photoelectron spectroscopy (UPS) was performed to investigate the interfacial energy level alignment after PDT modification. As shown in Figure S8 (Supporting Information), the valence band (VB) of PDT/PbI_2_ can be obtained from the equation of *E_VB_* = 21.22−(*E_cut‐off_* − *E_F,edge_*), and the conduction band (CB) can be calculated by E_CB_ = E_VB_ + E_g_. The E_g_ of PDT/PbI_2_ is obtained from the absorption spectra, as shown in Figure S9 (Supporting Information). Then, UPS was performed to measure the band structure of perovskite with and without MXene post treatment. As shown in Figure S10 (Supporting Information), the valance band (VB) of pristine and MXene modified perovskite films are calculated to be −5.46 and −5.52 eV, respectively. Based on these results, the energy diagram of the PSC is depicted in Figure 4b. The bottom PDT modification forms cascade energy level alignment, which facilitates the hole collection. Moreover, the high CB of PDT layer can function as electron block layer to suppress the charge recombination. On the other hand, the interaction between MXene and perovskite induces surface dipoles, leading to tuned interfacial band alignment.^[^ ^31^, ^38^ ^]^ The difference of band structure between the surface and bulk causes band‐bending,^[^ ^39^ ^]^ which is beneficial for charge extraction from perovskite to the PCBM layer. In short, the double‐sided interlayer modification forms favorable energy level alignment for efficient charge extraction and suppressed nonradiative charge recombination, which is beneficial for the enhanced photovoltaic performance.

The current‐voltage (*J‐V*) curves of the devices are shown in Figure 4c. The control device without modification shows *V_OC_* of 0.89 V, current density (*J_SC_*) of 26.04 mA cm^−2^, fill factor (FF) of 74.67%, and PCE of 17.31%. With the introduction of PDT interfacial layer, the photovoltaic performance increases obviously. At optimized conditions, the champion device demonstrates *V_OC_* of 0.93 V, *J_SC_* of 26.61 mA cm^−2^, FF of 77.36%, and PCE of 19.14%. The enhanced *V_OC_* and FF could be ascribed to the enhanced perovskite film quality with reduced defect density. With high PDT concentration, the *J_SC_* and FF show a serious drop, which comes from the increased internal series resistance due to the thick PDT layer. Then, MXene post treatment was introduced to further enhance the device performance. With the introduction of MXene interfacial layer, the champion device shows high PCE of 20.45% with *V_OC_* of 0.94 V, *J_SC_* of 27.83 mA cm^−2^ and FF of 78.17%. The much enhanced FF and *J_SC_* can be attributed to the enhanced charge extraction efficiency induced by the MXene interfacial layer. The statistical photovoltaic parameters are shown in Figure S11 (Supporting Information), which suggests the good reproducibility of the PDT/MXene treated device. The external quantum efficiency (EQE) curves of the control and PDT/MXene treated devices and corresponding integrated *J_SC_* are shown in Figure 4d, which aggress well with the values from the *J–V* curves.^[^ ^40^, ^41^ ^]^ Then, the environmental and light soaking stability of the ideal bandgap PSCs are examined. As shown in Figure 4e, the double‐sided interlayer modified PSC without encapsulation remained more than 85% of its initial efficiency after storage in N_2_ glove box for 1000 h. However, the control device degraded to ≈60% of its original efficiency under the same condition. Moreover, the light soaking stability of the device is also checked. As illustrated in Figure 4f, the double‐sided interlayer modified device without encapsulation maintained over 80% of the original efficiency after continuous white light illumination in N_2_ glovebox for 500 h. Nevertheless, the control device can only remain 50% of the initial efficiency under the same condition. The degradation of the device performance can be attributed to the oxidation of Sn^2+^ by the trace amount of oxygen in the glovebox. The enhanced device stability can be ascribed to the improved perovskite film quality with less defect density after PDT/MXene modification. In addition, the electron donor group from MXene can enhance the electron density of perovskite, leading to suppressed Sn^2+^ oxidation.^[^ ^42^, ^43^ ^]^ More importantly, 2D MXene flakes are impermeable to oxygen gas and ions, and consequently the inhibited ion migration in PSC with PDT/MXene interlayer contributed to the enhanced device stability.^[^ ^44^, ^45^, ^46^ ^]^

## 3 Conclusion

In summary, PDT was introduced as the bottom interlayer to enhance the performance of ideal bandgap Sn‐Pb mixed PSCs. The PDT spacer can act as a template to enhance the quality of the upper perovskite layer with reduced defect density. Meanwhile, the interlayer at the bottom interface is beneficial for the enhanced hole transfer and suppressed charge recombination. Then, Ti_3_C_2_T_x_ MXene was incorporated for surface treatment of perovskite to passivate the surface defects. The MXene treatment leads to the down shift of the energy band of the perovskite layer, which facilitates the interfacial charge transfer. Moreover, due to the improved quality of perovskite layer after the PDT treatment and the encapsulation of MXene layer on the top surface, the device stability was improved substantially. Beneficiating from these advantages, the ideal bandgap Sn‐Pb mixed PSCs show high PCE of 20.45% along with improved environmental and light soaking stability. This work opens new avenues for preparing high performance ideal bandgap PSCs.

## Conflict of Interest

The authors declare no conflict of interest.

## Supporting information

Supporting Information

## Data Availability

The data that support the findings of this study are available in the supplementary material of this article.
