# Supplementary material for: High‐Performance Ideal Bandgap Sn‐Pb Mixed Perovskite Solar Cells Achieved by MXene Passivation
Source: Small. 2024 Aug 15;20(47):2403920. doi: 10.1002/smll.202403920 (PMC11579958; doi:10.1002/smll.202403920)
Supplement: Supplementary file 1 — Supporting Information [file SMLL-20-2403920-s001.docx]

Supporting Information

**High-performance ideal bandgap Sn-Pb mixed perovskite solar cells achieved by MXene passivation**

*Jiupeng Cao^#^, Chun-ki Liu^#^, Yang Xu, Hok-Leung Loi, Tianyue Wang, Mitch Guijun Li, Lixian Liu, Feng Yan^*^*

J.P. Cao, C.-K. Liu, H.-L. Loi, T.Y. Wang, Prof. F. Yan*

Department of Applied Physics, The Hong Kong Polytechnic University

Hung Hom, Kowloon, Hong Kong SAR, P.R. China

E-mail: apafyan@polyu.edu.hk

Y. Xu, M. Li

Division of Integrative Systems and Design, Department of Electronic and Computer Engineering, The Hong Kong University of Science and Technology, Clear Water Bay, Kowloon, Hong Kong SAR, P.R.China

L.X. Liu

School of Optoelectronic Engineering, Xidian University, Xi’an 710071, China

Prof. F. Yan

Research Institute of Intelligent Wearable Systems, The Hong Kong Polytechnic University, Hung Hom, Kowloon, 999077, Hong Kong SAR, P.R. China

# Equal contribution

**Experimental section**

**Materials:** Formamidinium iodide (FAI) and methylammonium iodide (MAI) were obtained from Dyesol Ltd. Lead iodide (PbI_2_), tin iodide (SnI_2_), Cesium iodide (CsI) and tin fluorine (SnF_2_) were purchased from Alfa Aesar Inc. N,N-dimethylmethanamide (DMF), dimethyl-sulfoxide (DMSO) and diethyl ether were obtained from Sigma-Aldrich, Inc. Phenyl-C71-butyric acid methyl ester (PCBM) was purchased from Nano-C, Inc.

**Synthesis of MXene**: A modified minimally intensive layer delamination (MILD) synthesis method is used to prepare high quality MXene flakes. First, the etchant was prepared by mixing 0.4 g of LiF with 5mL of 9 M HCl. After that, 0.25 g of Ti_3_AlC_2_ was slowly added into the etchant. The reaction was allowed for 24 hours under continuous stirring. When the reaction was finished, the acidic mixture was washed with deionized water for multiple times until the pH value was near 7. During the washing cycles, acidic supernatant was decanted. The MXene flakes were then exfoliated by ultrasonication for 1 hour. The supernatant containing few-layer MXene was collected by centrifugation. The collected MXene water dispersion was dried and redispersed in isopropyl alcohol (IPA) (0.4 mg mL^-1^). Finally, the MXene in IPA dispersion was stored in a refrigerator for later use.

**Solar cell fabrication:** ITO glass substrates were ultrasonically cleaned by deionized (DI) water, acetone and IPA for 15 min, respectively. NiO_x_ dissolved in DI water (7.5 mg mL^-1^) was spin-coated on the ITO substrate at 4000 rpm for 30 s in ambient air and then the films were annealed at 150 ℃ for 30 min. Then the films were transferred to N_2_ glovebox. PDT/PbI_2_ (3/1) complex with different concentration was spin-coated on the NiO_x_ at 5000 rpm for 30s. The Sn-Pb mixed perovskite precursor was prepared by dissolving FAI (0.7 mmol), SnI_2_ (0.3mmol), SnF_2_ (0.03mmol), MAI (0.2 mmol), CsI (0.1 mmol) and PbI_2_ (0.7 mmol) in mixed solvent (DMF: DMSO=4:1). The perovskite precursor solution was spin-coated on the substrate at 1000 rpm for 10 s and 5000 rpm for 20 s, and diethyl ether was dropped on the film at 10^th^ s. The perovskite films were annealed at 70 ºC for 1 min and 100 ºC for 10 min. Then, MXene in IPA solution was spin-coated on the perovskite film at 3000 rpm for 30s. Afterward, PCBM (20 mg mL^-1^) and BCP (0.5 mg mL^-1^) were spin-coated on the films. Finally, 100 nm silver electrodes were thermally evaporated onto the films to fabricate PSCs with 0.1 cm^2^ active areas.

**Characterizations:** *J-V* curves were measured by a Keithley 2400 source meter with a solar simulator under AM 1.5 G one sun illumination (Newport 66902). The scanning rate was 100 mV s^-1^ with a voltage step of 10 mV, using both reverse (from V_OC_ to J_SC_) and forward (from J_SC_ to V_OC_) scans. The EQE of the PSCs was obtained from an EQE system under DC mode. UV-vis spectra were collected on a UV-vis spectrometer (Perkin elmer). SEM images were measured by a field-emission SEM (Tescan MAIA3). The TEM images were collected by JEOL JEM 2100F operated at 200 kV. XRD patterns were measured using Cu Kα radiation (Rigaku, Smartlab). XPS and UPS were measured on Thermo Fisher Scientific system. TOF SIMS V (ION-TOF GmbH) spectrometer was used for depth profile analysis. PL was measured by the FLS 920 (Edinburgh Instruments, Ltd).


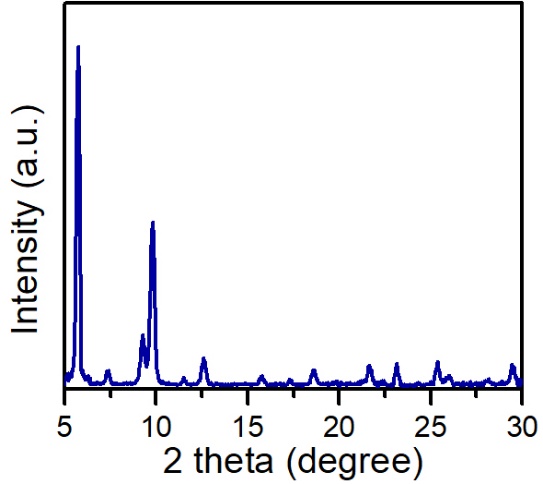


Figure S1. XRD pattern of PDT/PbI_2_ complex.


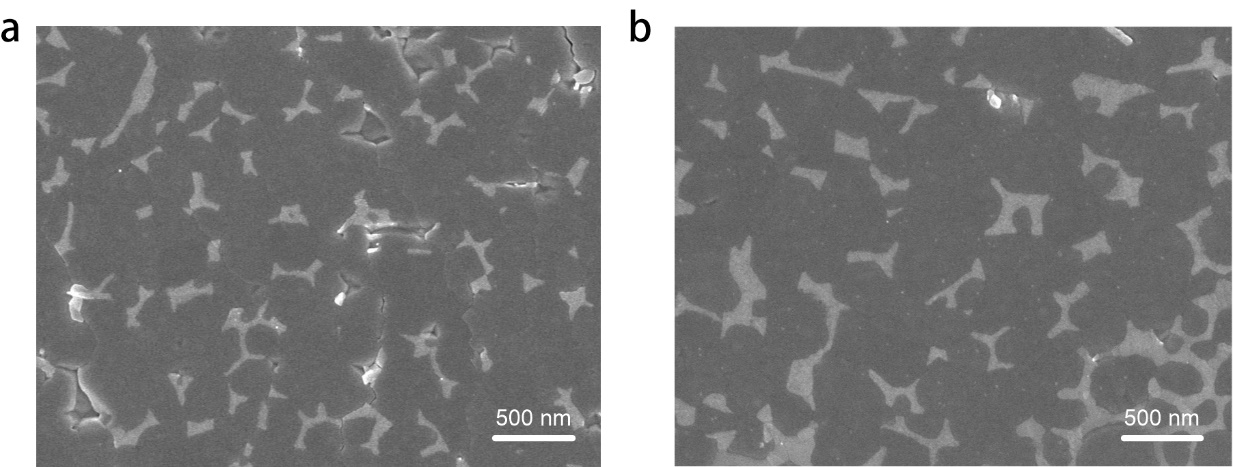


Figure S2. SEM images of the perovskite films from the bottom side (a) control, (b) with PDT interlayer.


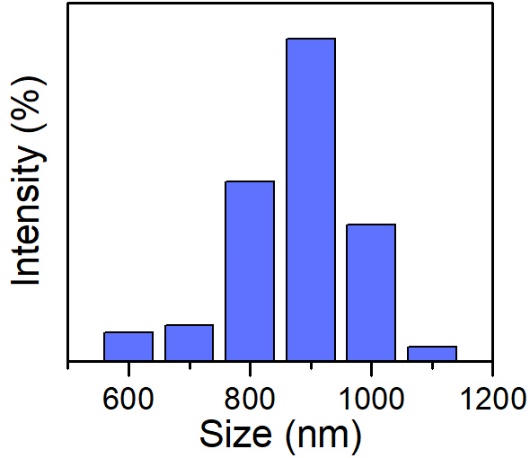


Figure S3. Size distribution of MXene nanosheets.


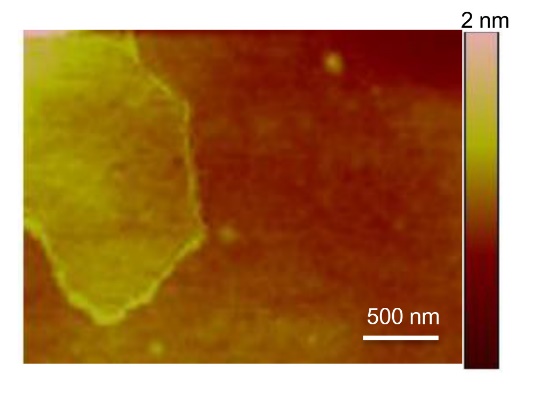


Figure S4. AFM image of MXene nanosheets.


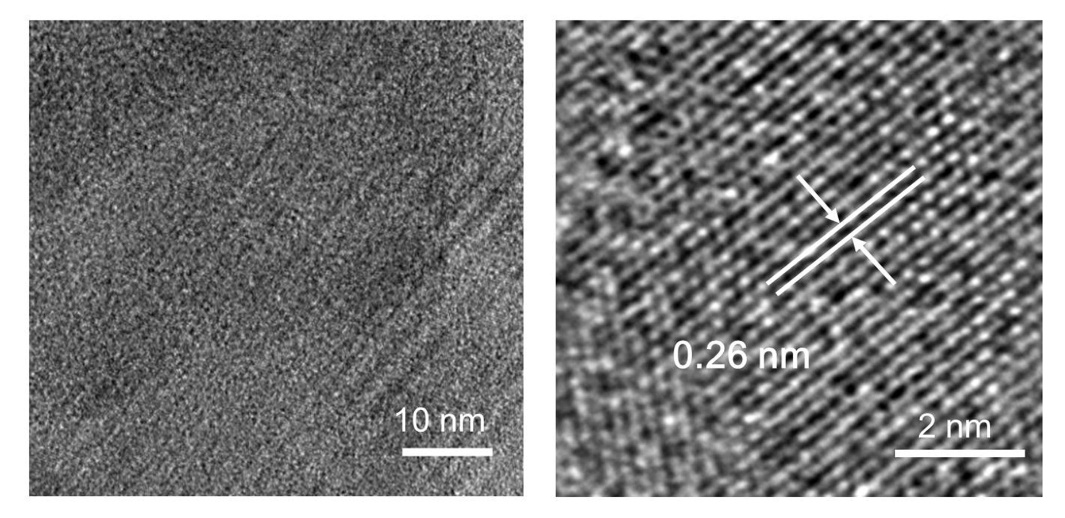


Figure S5. High resolution TEM images of MXene.


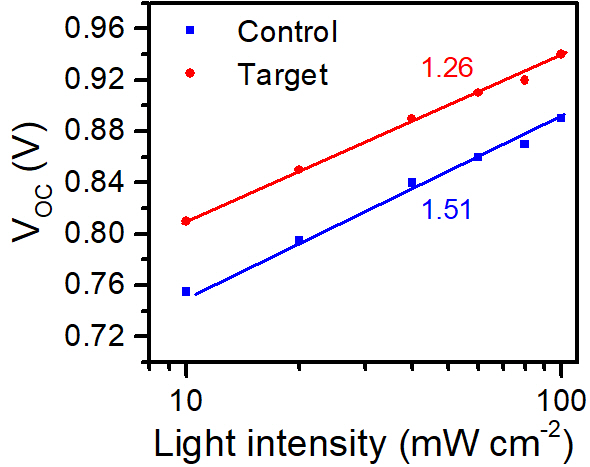


Figure S6. Light intensity dependence of V_OC_ for PSCs.


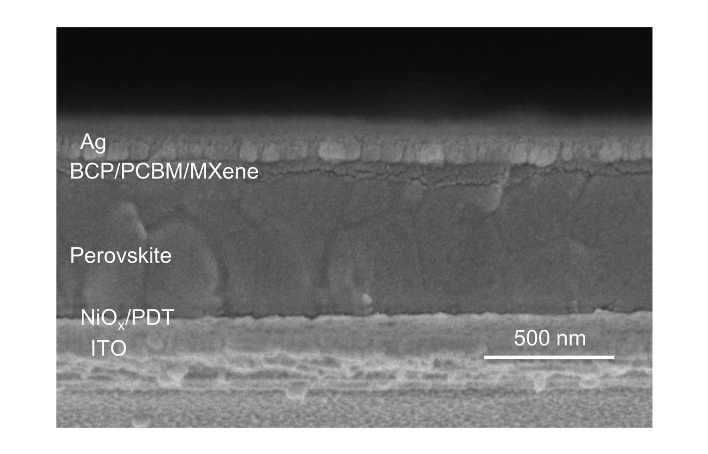


Figure S7. Cross sectional SEM image of the PSC.


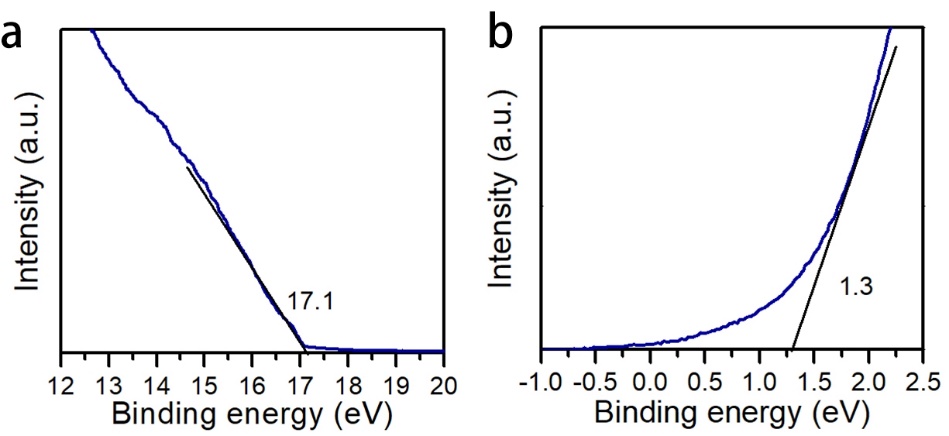


Figure S8. UPS spectra of the PDT/PbI_2_ complex.


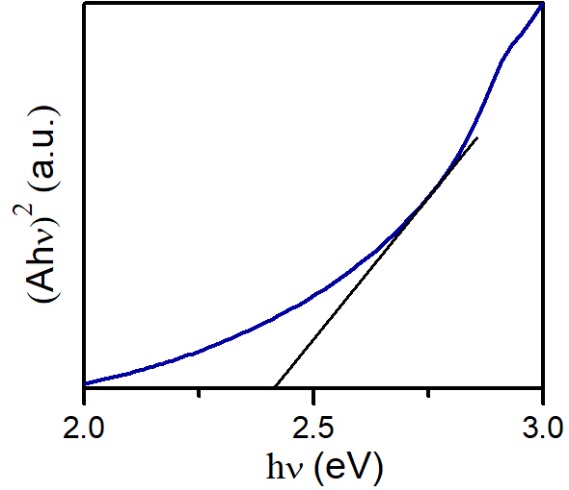


Figure S9. UV-vis spectra of the PDT/PbI_2_ complex.


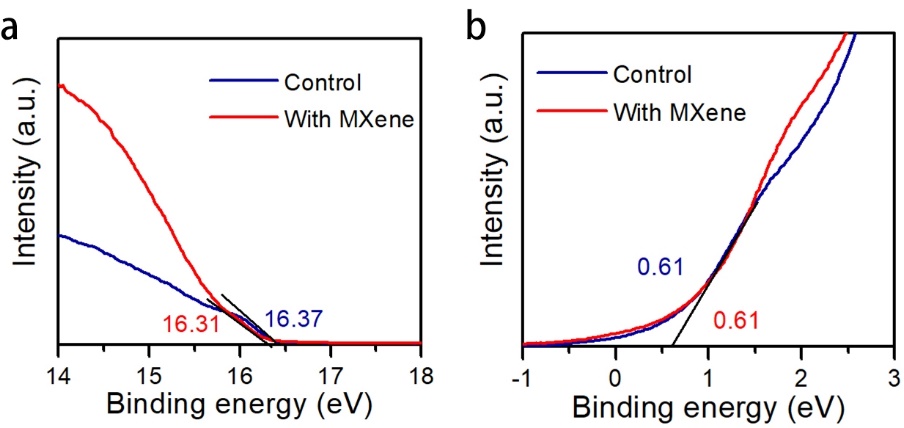


Figure S10. UPS spectra of the perovskite film without (a) and (b) with MXene modification.


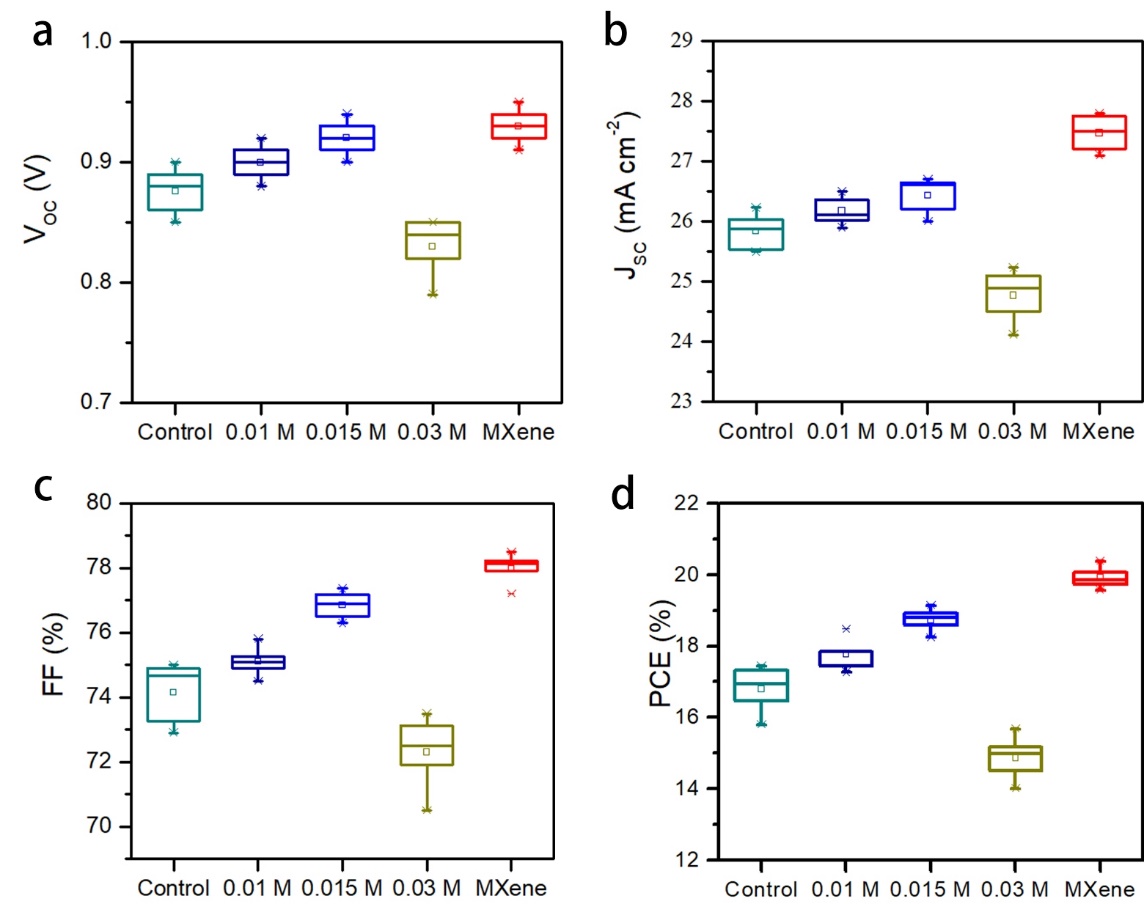


Figure S11. Statistical parameters of the PSCs.
